# Biliopancreatic Diversion (BPD), Long Common Limb Revisional Biliopancreatic Diversion (BPD + LCL–R), Roux-en-Y Gastric Bypass [RYGB] and Sleeve Gastrectomy (SG) mediate differential quantitative changes in body weight and qualitative modifications in body composition: a 5-year study

**DOI:** 10.1007/s00592-021-01777-9

**Published:** 2021-08-28

**Authors:** Valerio Ceriani, Ferdinando Pinna, Antonio Galantino, Ahmed S. Zakaria, Roberto Manfrini, Antonio E. Pontiroli, Franco Folli

**Affiliations:** 1 Dipartimento Di Chirurgia, IRCCS Multimedica, Milan, Italy; 2 Dipartimento Di Scienze Della Salute, Università Degli Studi Di Milano, Ospedale San Paolo, Via Antonio di Rudinì 8, 20142 Milan, Italy; 3 Endocrinologia E Malattie Metaboliche, Dipartimento Di Scienze Della Salute, Università Degli Studi Di Milano, Ospedale San Paolo, Via Antonio di Rudinì 8, 20142 Milan, Italy; 4 Unità Dipartimentale Di Diabetologia E Malattie Metaboliche, ASST Santi Paolo E Carlo, Milan, Italy

**Keywords:** Obesity, Bariatric surgery, Body composition, Fat mass, Fat-free mass, Total body water, Biliary pancreatic diversion, Revised biliary pancreatic diversion, Gastric bypass, Sleeve gastrectomy

## Abstract

**Aims:**

Bariatric surgeries induce profound weight loss (decrease in body mass index, BMI), through a decrease in fat mass (FM) and to a much lesser degree of fat-free mass (FFM). Some reports indicate that the weight which is lost after gastric bypass (RYGB) and sleeve gastrectomy (SG) is at least partially regained 2 years after surgery. Here we compare changes in BMI and body composition induced by four bariatric procedures in a 5 years follow-up study.

**Methods:**

We analyzed retrospectively modifications in BMI, FM and FFM obtained through Roux-en-Y gastric bypass (RYGB), sleeve gastrectomy (SG), biliopancreatic diversion (BPD) and a long common limb revisional biliopancreatic diversion (reduction of the gastric pouch and long common limb; BPD + LCL−R). Patients were evaluated at baseline and yearly for 5 years. Of the whole cohort of 565 patients, a subset of 180 patients had all yearly evaluations, while the remaining had incomplete evaluations. Setting University Hospital.

**Results:**

In a total of 180 patients evaluated yearly for 5 years, decrease in BMI and FM up to 2 years was more rapid with RYGB and SG than BPD and BPD + LCL−R; with RYGB and SG both BMI and FM slightly increased in the years 3–5. At 5 years, the differences were not significant. When analysing the differences between 2 and 5 years, BPD + LCL−R showed a somewhat greater effect on BMI and FM than RYGB, BPD and SG. Superimposable results were obtained when the whole cohort of 565 patients with incomplete evaluation was considered.

**Conclusions:**

All surgeries were highly effective in reducing BMI and fat mass at around 2 years; with RYGB and SG both BMI and FM slightly increased in the years 3–5, while BPD and BPD + LCL−R showed a slight further decreases in the same time interval.

**Supplementary Information:**

The online version contains supplementary material available at 10.1007/s00592-021-01777-9.

## Introduction

**Fig. 1:**
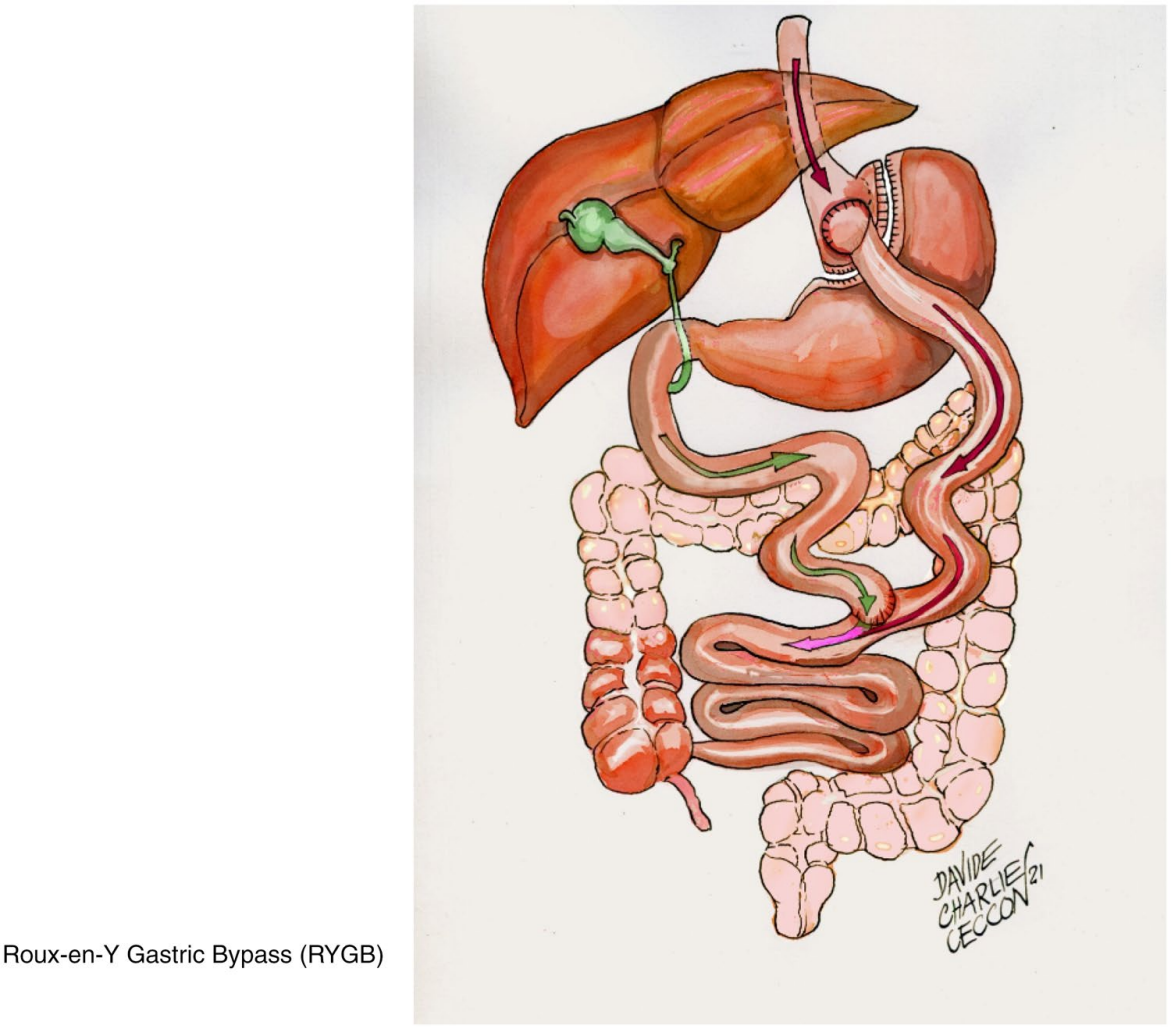
Roux-en-Y gastric bypass (RYGB). A small gastric pouch is created, to which a Roux-en-Y jejunal limb is anastomized. The larger portion of the stomach, excluded from transit of food, is typically left in place in the peritoneal cavity. The alimentary and bibliopancreatic limbs are not designed to establish a malabsorptive condition

**Fig. 2:**
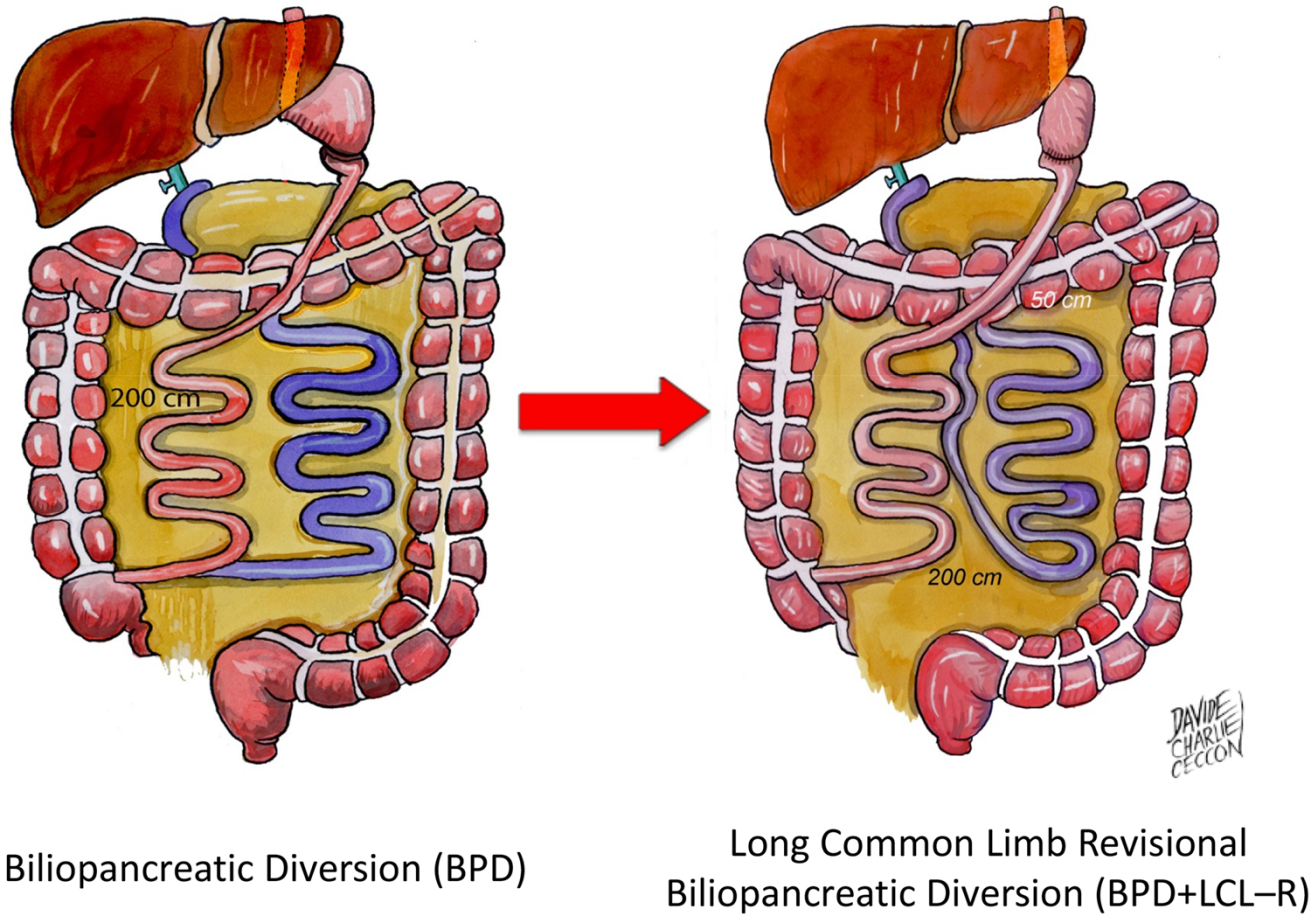
Biliopancreatic diversion with distal gastrectomy according to the original model by Scopinaro (BPD) on the left; long common limb revisional BPD (LCL-R BPD) on the right BPD is a malabsorptive procedure in which, by means of a distal gastrectomy and a long Roux-en-Y gastro-ileostomy, a 250-cm ileal limb is left in continuity and, along this, a 50-cm distal common channel is constructed, where the ingested nutrients could come in contact with biliopancreatic secretions. By diverting biliopancreatic secretions from contact with food along the majority of the small bowel, a condition of selective malabsorption for fat ensues, determining the appearance of a threshold for intestinal absorption of alimentary calories. LCL-R-BPD is graphically presented in close linkage with BPD, owing to direct anatomical and technical connections. From the original model of BPD, an elongationg of the common limb from 50 to 200 cm is performed, at the expense of the alimentary limb. Simultaneously, with the aim of avoiding weight regain, the gastric pouch is reduced from 500 to 40 ml. The resulting procedure consists of a gastric pouch of 40 ml and a total in continuity small bowel limb of 250 cm, with a common channel of 200 cm.

**Fig. 3:**
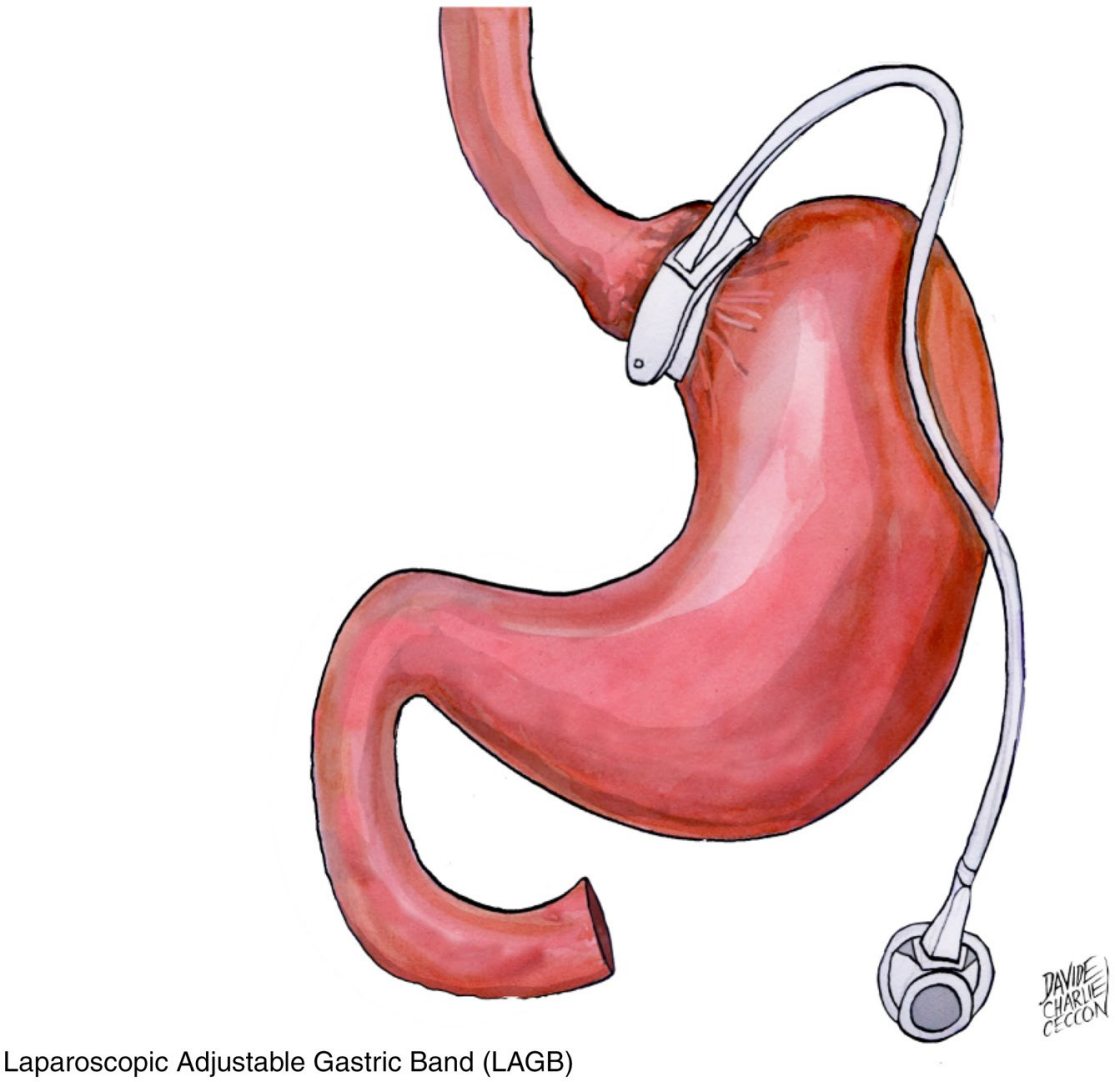
Laparoscopic adjustable gastric band (LAGB). A restrictive bariatric procedure, consisting in wrapping the subcardial region of the stomach with a silicone ring, which can be calibrated by injecting or removing fluid (e.g. sterile saline solution) from a connected subcutaneous port, thus regulating the width of the corresponding gastric lumen. In this way, a small upper gastric chamber is created, which empties through a narrow orifice in the remaining of the stomach, restricting the amount of food that could be introduced at a time

**Fig. 4:**
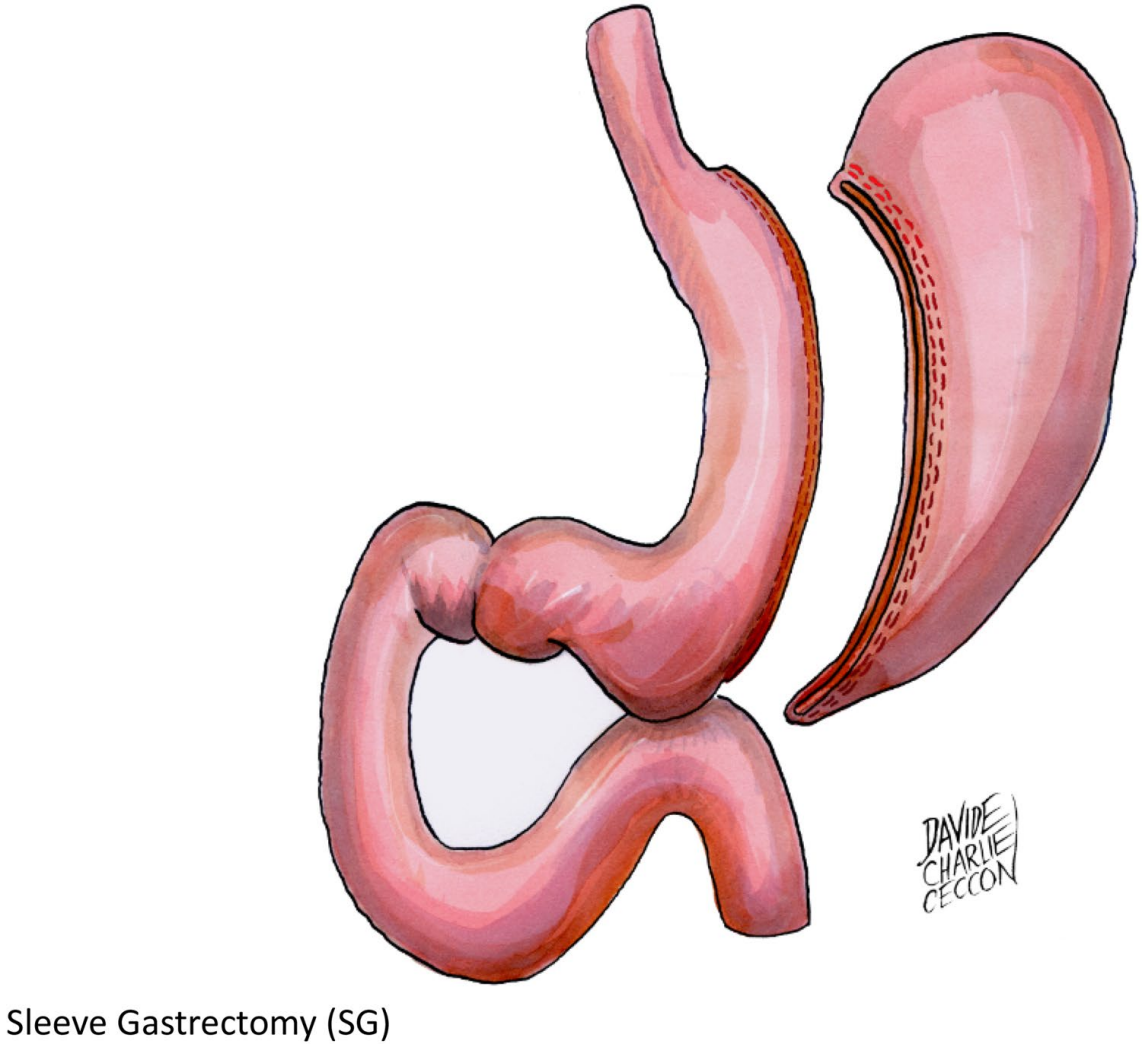
Sleeve gastrectomy (SG). A restrictive bariatric procedure in which the gastric fundus and the majority of the gastric body are removed, leaving in place the pyloric antrum and a narrow gastric tubule with the shape of a sleeve in continuity with the oesophagus. The procedure determines a reduction of the gastric capacity to approximately 15–25% of the starting values

Bariatric surgeries have increased progressively during the last 25 years, and currently, Italy stands among the five leading countries for number of procedures performed in the last 5 years [1]. There is consensus that gastric bypass (RYGB) (Fig. 1) and biliopancreatic diversion (BPD) (Fig. 2 on the left) are the most effective, as compared to gastric band (LAGB) (Fig. 3) for percent excess weight loss (EWL%), duration of weight loss and for resolution of co-morbidities [2, 3]. Sleeve gastrectomy (SG) (Fig. 4) is somehow similar to RYGB, both in terms of EWL%, or percent decrease in body mass index (BMI, %EBL) and resolution of co-morbidities [4–11]. One of the most important co-morbidities in which bariatric surgery has a positive impact is type 2 diabetes mellitus (DM2), because the weight loss represents a key element in correcting the metabolic alterations of the obese subject with DM2 [12]. In particular, the abdominal visceral fat excess plays a significant role because induces and maintains lipotoxicity and insulin resistance with increased risk of macrovascular complications [13, 14]. In these subjects, it is difficult/impossible to achieve a significant and lasting weight loss through medical treatment [15]. In addition, drugs such as sulfonylureas, glitazones and insulin employed in DM2 hinder weight loss, making the goal of effective weight reduction even more difficult [16]. In this context, bariatric/metabolic surgery has proven to be an effective option in morbidly obese subjects with DM2 failing with conventional diets and in many cases has even resulted in the clinical remission of the hyperglycemia [17]. A constellation of factors is likely responsible for the improvement or resolution of DM2 following metabolic surgery. Among these are noteworthy the enhancement of the neural signalling, the changes in gut hormones release (GLP1 and GIP), the modulation of the intestinal microbioma and bile composition and the acute reduction of glucoxicity/lipotoxicity [18, 19]. These factors improve beta cells function and insulin sensitivity with achievement of optimal control or remission of DM2 [20, 21]. Studies with a 4 years follow-up have confirmed the similarity between RYGB and SG, and superiority of BPD vs both RYGB and SG in terms of %EBL [22–24]. In addition, surgical revision of BPD through reduction of the gastric pouch and elongation of the common limb (long common limb revisional BPD, BPD + LCL−R) (Fig. 2 on the right), performed because of complications or of insufficient weight loss, has demonstrated a significant further decrease in BMI [25–27] over the classical Scopinaro BPD. However, longitudinal studies have shown that the effect of both RYGB and SG on %EBL decreases slightly after the first-year post-surgery, while BPD seems to maintain its effects on %EBL. Similar results have been shown in a few studies for the decrease in fat mass [6, 7, 11, 22–24, 28, 29]. The aim of this study was to compare longitudinally the effect of BPD, RYGB, SG and BPD + LCL−R on %EBL and body composition for 5 years post-surgery in subjects operated in the same Institution.

## Subjects and methods

This is a retrospective study on a cohort of 565 consecutive obese subjects undergoing bariatric surgery at Istituto Multimedica, Milan, Italy between 2010 and 2014. All subjects were operated by the same surgeons (V.C. and F.P.). BPD has been performed since 2002, employing the Scopinaro technique [30]; RYGB has been performed since 2003 and SG since 2010. The revised BPD surgery (BPD + LCL−R) was introduced in 2007 to reduce severe side effects and complications of BPD [25–27]. It consists of gastric reduction to 40 ml and elongation of the common limb to 200 cm at the expenses of the alimentary limb. Indications for revisional surgery were various: anorectal complications, malabsorption, malnutrition as well as insufficient weight loss. The choice of bariatric surgery technique was discussed with the patients, based in particular on their preferences and expectations, and BMI; following the guidelines on metabolic and bariatric surgery in the period spanning from 2000 to 2010 subjects with more severe obesity underwent purely malabsorptive procedures (BPD and possibly revision to LCL-R according to clinical requirements) rather than RYGB or SG. At all scheduled visits [baseline and at each year for 5 years], patients were evaluated for BMI, and for body composition. BMI was calculated as weight/square height [kg/m^2^]; body composition was measured by body impedance analysis (BIA), through a 8 electrodes Tanita BC 418 (Tanita Impedance Balance, Tokyo, Japan) to assess fat mass (FM, kg and as percentage), fat-free mass (FFM, kg and as percentage) and total body water (TBW, absolute and percentage). Several studies have supported the validity of BIA in obese and non-obese patients [31–35]. Weight loss was calculated as percent BMI loss (%EBL) [36].

## Statistical analysis

Data are presented as means ± SD in Tables and as means ± SE in Figures. For each bariatric technique, BMI, %EBL, FM, FFM and TBW were analyzed at each time interval by one-way analysis of variance (ANOVA). In addition, based on several reports of decrease in efficacy of RYGB and SG on %EBL and body composition from one-year post-op onwards [6, 37, 38], the difference between values registered at 2 and 5 years was also calculated. Since a high proportion of subjects was unavailable after 3 years, we performed two analyses, one for the whole cohort of 565 subjects, the other for the 180 subjects who attended the annual body composition assessment for 5 years. In the text, data analysis included 180 subjects who performed an annual over a five-year period; in the supplementary appendix, data analysis included all 565 subjects. All statistical analyses were performed employing Stata 12 for Macintosh (Stata Corporation, College Station, Texas).

## Results

**Fig. 5:**
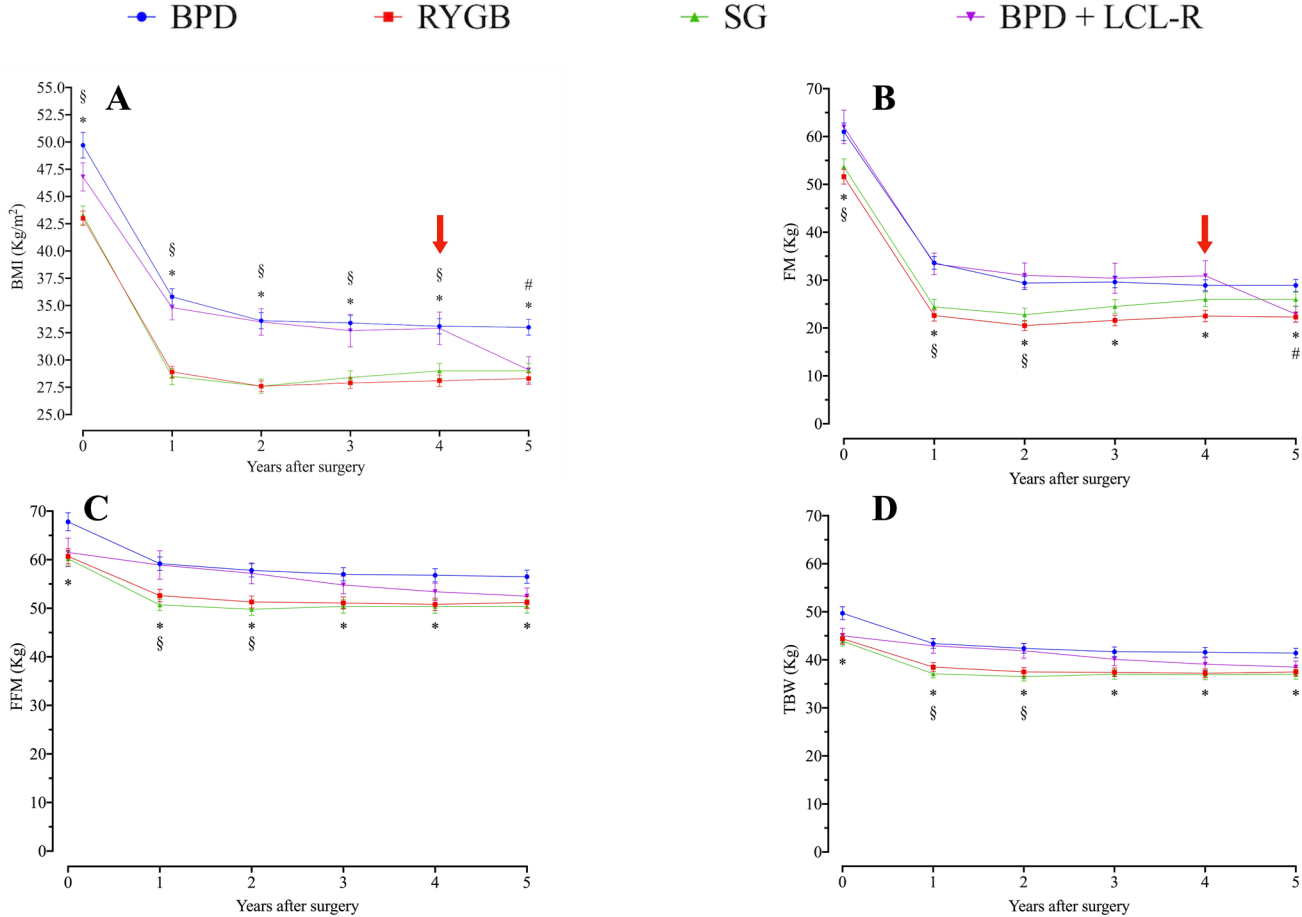
Body mass index (BMI), fat mass (FM), fat-free mass (FFM) and total body water (TBW) at baseline and at yearly visits up to 5 years in subjects undergoing the four kinds of bariatric surgery. Means ± SE. The red arrow indicates the median time for revisional surgery in subjects undergoing BPD + LCL−R

**Fig. 6:**
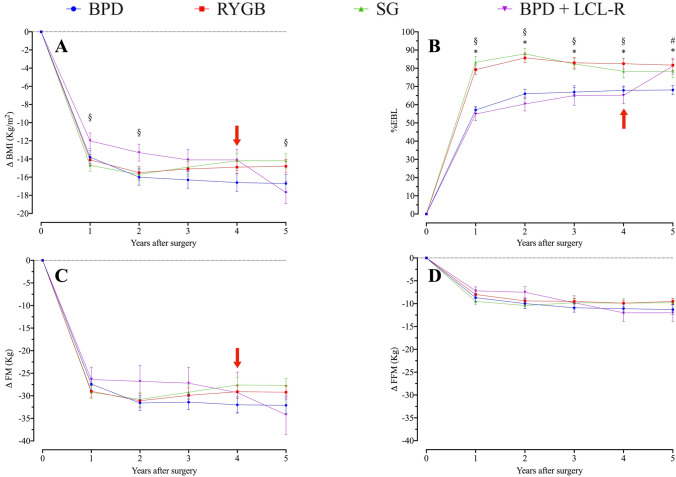
Excess body mass loss (%EBL), and change of BMI, FM, FFM at baseline and at yearly visits up to 5 years in subjects undergoing the four kinds of bariatric surgery. Means ± SE

**Fig. 7:**
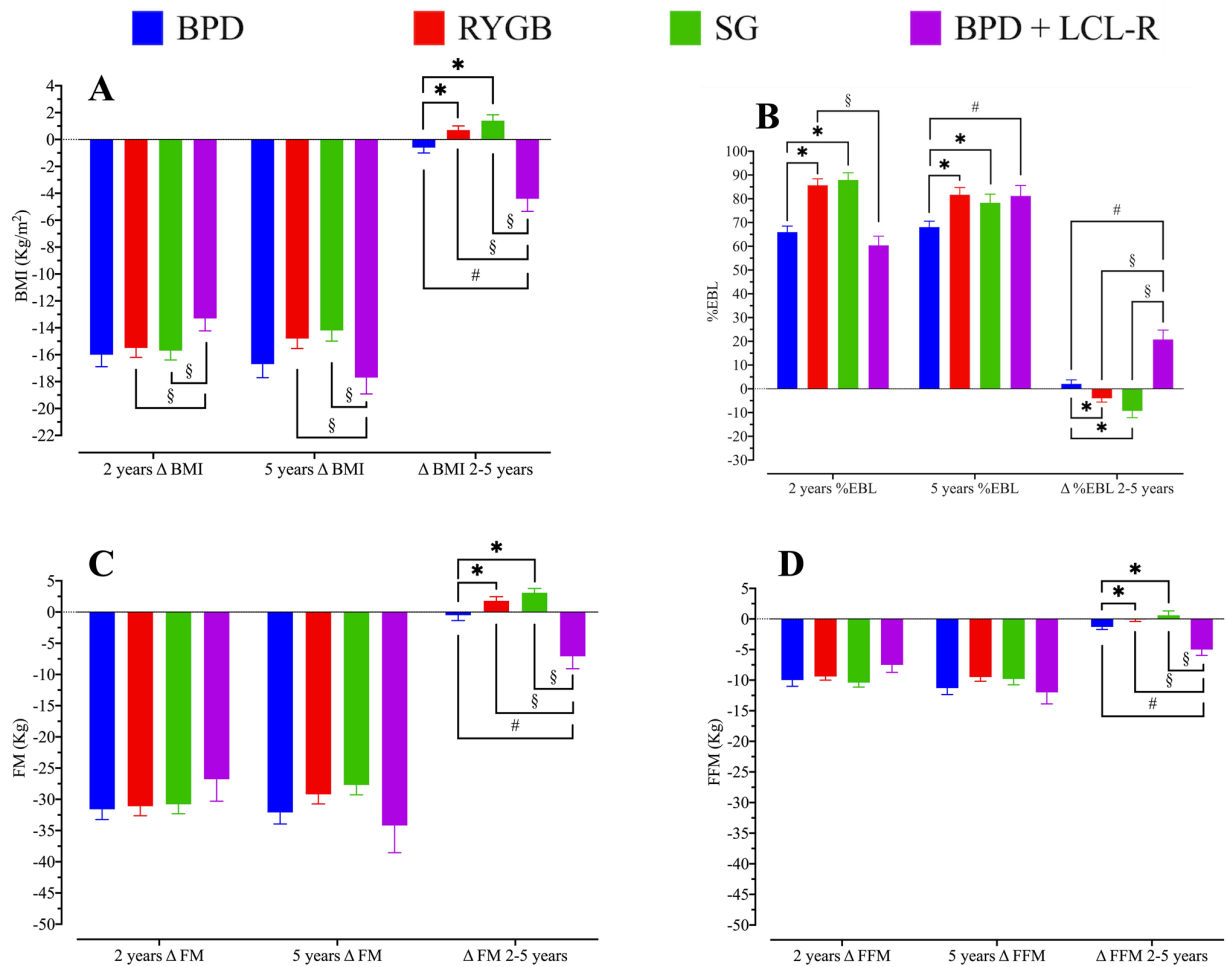
Changes of %EBL, BMI, FM and FFM observed at 2 years, 5 years, and between 2 and 5 years after the four kinds of bariatric surgery. Means ± SE

Surgical techniques (BPD and BPD + LCL−R) have been previously described [25, 26, 30]. Table 1 shows body composition of 180 patients evaluated at all time intervals, and Supplemental Appendix Table 1 shows body composition of total cohort at baseline (*n* = 565). BMI was different at baseline, and changes thereafter were somewhat parallel, but BPD + LCL−R showed a further decrease at 5 years, reaching values of RYGB and SG at the end of the observation period (Fig. 5a). In the five-years follow-up, RYGB and SG showed a peak of BMI and FM loss at two years of follow-up  followed by a slight increase in subsequent years of observation (Fig. 5a, b). A similar trend was observed for fat mass (FM) (Fig. 5b). Also, fat-free mass (FFM) showed an analogous trend, with less marked differences among single surgeries (Fig. 5c). Finally, TBW showed a trend superimposable to FFM (Fig. 5d). With RYGB and SG, the decrease in both BMI and FM peaked at 1–2 years, with slight increases thereafter up to 5 years. This behaviour was not seen with BPD and BPD + LCL−R (Fig. 5a, b). Changes of BMI were similar for BPD, RYGB and SG (Δ BMI, Fig. 6a). BPD + LCL−R showed a different trend, with a slower decrease for the first 2 years and greater decrease at 5 years compared to RYGB, SG and BPD (Δ BMI, Fig. 6a). %EBL showed a more rapid increase in RYGB and SG than in BPD and BPD + LCL−R, with somehow parallel trend thereafter, greater for BPD + LCL−R, so that at 5 years, values were similar for BPD + LCL−R, RYGB and SG, and slightly greater than BPD (Fig. 6b). Changes of FM and FFM (Δ FM, Δ FFM) were not different among the four techiques (Fig. 6c, d). Figure 6a–d shows the differences between surgical techniques at each time interval. Differences in the four techniques between 2 and 5 years in reduction of BMI, FM and FFM and increase of %EBL were all slightly greater with BPD + LCL−R as compared to BPD, RYGB and SG. Also, for Δ BMI, Δ %EBL and Δ FM, the change peaked at 1–2 years, with a slight decrease thereafter; this pattern was not seen with BPD + LCL−R, which showed a slight further decrease (Fig. 7).

**Table 1:** Details of patients undergoing bariatric surgery at baseline and evaluated yearly for 5 years. Absolute numbers and Means ± SD

	BPD	RYGB	SG	BPD + LCL−R	Significance (*p*)
Number (M/W)	56 (12/44)	48 (8/40)	46 (6/40)	30 (6/24)	NS
Age (years)	45.2 ± 10.98	44.5 ± 10.29	43.5 ± 11.40	42.7 ± 10.86	NS
BMI (kg/m^2^)	49.7 ± 8.76	43.1 ± 4.57*	43.3 ± 5.68*§	46.8 ± 7.01	0.001
Weight (kg)	128.7 ± 23.94	112.2 ± 16.02*	113.5 ± 16.94*§	125.0 ± 23.09	0.001
Excess weight (kg)	63.5 ± 22.41	47.1 ± 12.47*	47.9 ± 14.74*§	59.9 ± 18.86	0.001
FM (kg)	61.0 ± 13.84	51.6 ± 10.64*§	53.7 ± 11.16*	62.0 ± 9.94	0.001
FFM (kg)	67.8 ± 13.85	60.7 ± 10.82*	60.2 ± 10.11*	61.5 ± 8.32	0.003
FM (%)	47.3 ± 5.42	45.9 ± 5.74	47.1 ± 4.85	50.1 ± 4.26	NS
FFM (%)	52.8 ± 5.27	54.1 ± 5.74	53.2 ± 5.17	49.9 ± 4.26	NS
TBW (%)	49.7 ± 10.28	44.4 ± 7.91*	43.9 ± 6.92*	45.0 ± 6.10	0.002

BPD = biliopancreatic diversion; RYGB = gastric bypass; SG = sleeve gastrectomy; BPD + LCL−R = biliopancreatic diversion followed by elongation of the common limb and restriction of the gastric pouch (median interval 4 years) FM = fat mass; FFM = fat-free mass; TBW = total body water

* versus BPD and BPD + LCL−R; § versus RYGB

The Supplemental Appendix reports data of all 565 patients undergoing bariatric surgery; changes of BMI, FM, FFM, TBW and %EBL were superimposable to those observed in the 180 patients reported above. In addition, supplemental Fig. 3 shows data obtained in the current whole cohort in comparison with historical data of BPD and BPD + LCL−R (redrawn from references 26, 27). The 180 subjects completing the 5-year observation period and the remaining 385 subjects were compared; there was no significant difference between the two groups was observed for age, sex distribution, initial BMI, FM, FFM, %EWL, %FM, %FFM and TBW (not shown). Sex differences for baseline conditions and for changes from year 1 are shown in the Supplementary Appendix, for both the 180 subjects and the full cohort (Tables 7–18). Women, in spite of similar BMI, had greater fat mass and lower fat-free mass and TBW than men. Change of BMI was similar in men and women; decrease in fat mass, in fat-free mass and in TBW was greater in men than in women, although differences present at baseline were maintained.

## Discussion

In this study, we compared changes of BMI and of fat mass obtained  up to 5 years after surgery in patients undergoing BPD, RYGB, SG and a revisional BPD procedure (BPD + LCL−R). The possible short- or long-term complications of the different surgeries were not reported here because our focus was to describe differential changes in body composition. As previously reported, in RYGB and SG, the initial fast reduction in BMI and FM reached a plateau from years 1–2 to 5 years [6, 7, 11, 22–24, 28, 29]. This pattern was not observed with BPD and BPD + LCL−R, after which there was a further slight progressive decrease in both BMI and FM. Also, the differences between 2nd year and 5th year were greater for BPD + LCL−R than for the three other techniques. These differences were observed both in the whole cohort and in the 180 patients evaluated each year for 5 years.

Attenuation of the initial effect on BMI and FM has repeatedly been described also for dietetic interventions and for lifestyle modification interventions [39, 40] and is known. Additional factors that could affect weight loss after bariatric surgery have been identified in the past, such as ethnic group [39], initial BMI, age, compliance to post-surgery diet and scheduled visits [40–45].

Loss of FFM seems to be an obligate side-effect of all bariatric surgeries and of rapid weight losses, although it is usually greatly inferior to FM loss. In this study, up to 5 years, FFM loss was similar (not exceeding 10kg) with all bariatric surgeries. There has been discussion on the effects of FFM loss on well being and strength, but it has been recently shown that well being and general strength are not compromised by weight loss after bariatric surgery [46].

Data from previous studies show that similar to gastric bypass and LAGB, the effect of BPD on glucose metabolism ranged from reduction of beta cell toxicity and apoptosis with increased insulin sensitivity to pro-incretinic effect [47–53]. On the other hands, BPD has shown to reverse the major components of the metabolic syndrome for at least 10 years in subjects with severe obesity, but showed less efficacy in inducing remission of diabetes in overweight or non-morbid DM2 obesity subjects [54, 55].

Our study, conducted in a morbid obesity population, shows that positive changes in body composition seem more stable and durable with BPD than with other bariatric surgery techniques. Given the preponderant role that BMI and fat mass have in determining and maintaining DM2, BPD and BPD + LCL−R should always be considered an option to achieve a progressive and sustained weight loss over time also in diabetic subjects with severe obesity and insulin resistance failing with the usual medical and nutritional therapy.

## Limitations

This is a retrospective study, with surgeries performed in different periods (BPD since 2002, RYGB since 2003, BPD + LCL−R since 2007 and SG since 2010). In addition, candidates were suggested different surgeries based on the attitudes of the years 2000–2010, and therefore, heavier patients preferentially received BPD and BPD + LCL−R, rather than RYGB or (later) SG. Finally, only 180 out of 565 subjects had a yearly visit, comprehensive of body composition, for 5 years; this latter aspect is partly overcome by the fact that in terms of weight loss, the results obtained in the two series of subjects were virtually identical.

## Conclusions

With RYGB and SG, there is somewhat more rapid decrease in BMI and of FM than with BPD and BPD + LCL−R. During the following period, from year 2 to year 5, there is partial loss of effect on BMI and on FM for RYGB and SG, while loss of effect is not seen in BPD and BPD + LCL−R. At 5 years, BPD + LCL−R, compared to RYGB, SG and BPD, produces a further slight reduction of BMI, fat mass and fat-free mass. Longer observation periods in multicenter studies are necessary to corroborate these findings.

## Supplementary Information

Below is the link to the electronic supplementary material.

Supplementary file1 (DOCX 1133 KB)

## References

[1] Welbourn R, Hollyman M, Kinsman R (2019). Bariatric surgery worldwide: baseline demographic description and one-year outcomes from the Fourth IFSO global registry report 2018. Obes Surg.

[2] Buchwald H, Avidor Y, Braunwald E (2004). Bariatric surgery: a systematic review and meta-analysis. JAMA.

[3] Folli F, Pontiroli AE, Schwesinger WH (2007). Metabolic aspects of bariatric surgery. Med Clin North Am.

[4] Zhang C, Yuan Y, Qiu C, Zhang W (2014). A meta-analysis of 2-year effect after surgery: laparoscopic Roux-en-Y gastric bypass versus laparoscopic sleeve gastrectomy for morbid obesity and diabetes mellitus. Obes Surg.

[5] Kang JH, Le QA (2017). Effectiveness of bariatric surgical procedures: a systematic review and network meta-analysis of randomized controlled trials. Medicine (Baltimore)..

[6] Salminen P, Helmiö M, Ovaska J (2018). Effect of laparoscopic sleeve gastrectomy vs laparoscopic roux-en-y gastric bypass on weight loss at 5 years among subjects with morbid obesity: The SLEEVEPASS randomized clinical trial. JAMA.

[7] Silva LB, Oliveira BMPM, Correia F (2019). Evolution of body composition of obese subjects undergoing bariatric surgery. Clin Nutr ESPEN.

[8] Bettencourt-Silva R, Neves JS, Pedro J (2019). Comparative Effectiveness of Different Bariatric Procedures in Super Morbid Obesity. Obes Surg.

[9] Shivakumar S, Tantia O, Goyal G (2018). LSG vs MGB-OAGB-3 year follow-up data: a randomised control trial. Obes Surg.

[10] McTigue KM, Wellman R, Nauman E (2020). Comparing the 5-year diabetes outcomes of sleeve gastrectomy and gastric bypass: the national patient-centered clinical research network (PCORNet) bariatric study. JAMA Surg.

[11] Oliveira SC, Neves JS, Souteiro P (2020). Impact of bariatric surgery on long-term cardiovascular risk: comparative effectiveness of different surgical procedures. Obes Surg.

[12] Brown SA, Upchurch S, Anding R, Winter M, Ramìrez G (1996). Promoting weight loss in Type II diabetes. Diabetes Care.

[13] Gastaldelli A, Miyazaki Y, Pettiti M (2002). Metabolic effects of visceral fat accumulation in type 2 diabetes. J Clin Endocrinol Metab.

[14] Ginsberg HN (2000). Insulin resistance and cardiovascular disease. J Clin Invest.

[15] Xavier Pi-Sunyer F (2005). Weight loss in type 2 diabetic subjects. Diabetes Care.

[16] Cheng V, Kashyap SR (2011). Weight considerations in pharmacotherapy for Type 2 diabetes. J Obes.

[17] Thaler JP, Cummings DE (2009). Minireview: hormonal and metabolic mechanisms of diabetes remission after gastrointestinal surgery. Endocrinology.

[18] Kamvissi-Lorenz V, Raffaelli M, Bornstein S (2017). Role of the gut on glucose homeostasis: lesson learned from metabolic surgery. Curr Atheroscler Rep.

[19] Batterham RL, Cummings DE (2016). Mechanisms of diabetes improvement following bariatric/metabolic surgery. Diabetes Care.

[20] Rubino F, Schauer PR, Kaplan LM, Cummings DE (2010). Metabolic surgery to treat type 2 diabetes: clinical outcomes and mechanisms of action. Ann Rev Med.

[21] Schauer PR, Mingrone G, Ikramuddin S, Wolfe B (2016). Clinical outcomes of metabolic surgery: efficacy of glycemic control, weight loss, and remission of diabetes. Diabetes Care.

[22] Strain GW, Gagner M, Pomp A (2009). Comparison of weight loss and body composition changes with four surgical procedures. Surg Obes Relat Dis.

[23] Risstad H, Kristinsson JA, Fagerland MW (2017). Bile acid profiles over 5 years after gastric bypass and duodenal switch: results from a randomized clinical trial. Surg Obes Relat Dis.

[24] Elias K, Bekhali Z, Hedberg J, Graf W, Sundbom M (2018). Changes in bowel habits and patient-scored symptoms after Roux-en-Y gastric bypass and biliopancreatic diversion with duodenal switch. Surg Obes Relat Dis.

[25] Ceriani V, Cetta F, Pinna F, Pontiroli AE (2016). Abnormal calcium, 25(OH)vitamin D, and parathyroid hormone after biliopancreatic diversion; correction through elongation of the common tract and reduction of the gastric pouch. Surg Obes Relat Dis.

[26] Ceriani V, Pinna F, Lodi T, Pontiroli AE (2017). Revision of biliopancreatic diversion for side effects or insufficient weight loss: codification of a new procedure. Obes Surg.

[27] Ceriani V, Cetta F, Lodi T, Pinna F, Pontiroli AE (2017). Clinical and Metabolic effects of biliopancreatic diversion persist after reduction of the gastric pouch and elongation of the common alimentary tract. preliminary report in a series of subjects with a 10-year follow-up. Obes Surg.

[28] Maciejewski ML, Arterburn DE, Scoyoc L (2016). Bariatric surgery and long-term durability of weight loss. JAMA Surg.

[29] Sherf-Dagan S, Zelber-Sagi S, Buch A (2019). Prospective longitudinal trends in body composition and clinical outcomes 3 years following sleeve gastrectomy. Obes Surg.

[30] Scopinaro N, Adami GF, Marinari GM (1998). Biliopancreatic diversion. World J Surg.

[31] Pontiroli AE, Folli F, Paganelli M (2005). Laparoscopic gastric banding prevents type 2 diabetes and arterial hypertension and induces their remission in morbid obesity: a 4-year case-controlled study. Diabetes Care.

[32] Frigé F, Laneri M, Veronelli A (2009). Bariatric surgery in obesity: changes of glucose and lipid metabolism correlate with changes of fat mass. Nutr Metab Cardiovasc Dis.

[33] Widen EM, Strain G, King WC (2014). Validity of bioelectrical impedance analysis for measuring changes in body water and percent fat after bariatric surgery. Obes Surg.

[34] Achamrah N, Colange G, Delay J (2018). Comparison of body composition assessment by DXA and BIA according to the body mass index: a retrospective study on 3655 measures. PLoS One..

[35] Beato GC, Ravelli MN, Crisp AH, de Oliveira MRM (2019). Agreement between body composition assessed by bioelectrical impedance analysis and doubly labeled water in obese women submitted to bariatric surgery : body composition, BIA, and DLW. Obes Surg.

[36] Deitel M, Gawdat K, Melissas J (2007). Reporting weight loss 2007. Obes Surg.

[37] Belligoli A, Bettini S, Segato G, Busetto L (2020). Predicting responses to bariatric and metabolic surgery. Curr Obes Rep.

[38] Cornejo-Pareja I, Molina-Vega M, Gómez-Pérez AM, Damas-Fuentes M, Tinahones FJ (2021). Factors related to weight loss maintenance in the medium-long term after bariatric surgery: a review. J Clin Med.

[39] Fazliana M, Liyana AZ, Omar A (2018). Effects of weight loss intervention on body composition and blood pressure among overweight and obese women: findings from the MyBFF@home study. BMC Womens Health.

[40] Seimon RV, Wild-Taylor AL, Keating SE (2019). Effect of weight loss via severe vs moderate energy restriction on lean mass and body composition among postmenopausal women with obesity: the TEMPO diet randomized clinical trial. JAMA Netw Open.

[41] Wood MH, Carlin AM, Ghaferi AA (2019). Association of race with bariatric surgery outcomes. JAMA Surg.

[42] Pontiroli AE, Fossati A, Vedani P (2007). Post-surgery adherence to scheduled visits and compliance, more than personality disorders, predict outcome of bariatric restrictive surgery in morbidly obese subjects. Obes Surg.

[43] Wood GC, Benotti PN, Lee CJ (2016). Evaluation of the association between preoperative clinical factors and long-term weight loss after Roux-en-Y gastric bypass. JAMA Surg.

[44] Sillén L, Andersson E (2017). Patient factors predicting weight loss after Roux-en-Y gastric bypass. J Obes.

[45] Carey DG, Raymond RL (2008). Can body mass index predict percent body fat and changes in percent body fat with weight loss in bariatric surgery subjects?. J Strength Cond Res.

[46] Alba DL, Wu L, Cawthon PM (2019). Changes in lean mass, absolute and relative muscle strength, and physical performance after gastric bypass surgery. J Clin Endocrinol Metab.

[47] Hinlst JJ (2013). Enteroendocrine secretion of gut hormones in diabetes, obesity and after bariatric surgery. Curr Opin Pharmacol.

[48] Ferrannini E, Mingrone G (2009). Impact of different bariatric surgical procedures on insulin action and –cell function in type 2 diabetes. Diabetes Care.

[49] Pontiroli AE, Pizzocri P, Librenti MC (2002). Laparoscopic adjustable gastric banding for the treatment of morbid (grade 3) obesity and its metabolic complications: a three-year study. J Clin Endocrinol Metab.

[50] Pontiroli AE, Folli F, Paganelli M (2005). Laparoscopic gastric banding prevents type 2 diabetes and arterial hypertension and induces their remission in morbid obesity. A 4-year case-controlled study. Diabetes Care.

[51] Veronelli A, Laneri M, Ranieri R (2004). White blood cells in obesity and diabetes. Effects of weight loss and normalization of glucose metabolism. Diabetes Care.

[52] Pontiroli AE, Zakaria AS, Micheletto G (2019). A 9 years comparison of weight loss, disappearance of obesity, and resolution of diabetes mellitus with biliointestinal bypass and with adjustable gastric banding: experience of a collaborative network. Acta Diabetologica.

[53] Gentileschi P, Bianciardi E, Benavoli D, Campanelli M (2021). Metabolic surgery for type II diabetes: an update. Acta Diabetol.

[54] Scopinaro N, Marinari GM, Camerini GB, Papadia FS, Adami GF (2005). Specific Effects of biliopancreatic diversion on the major components of metabolic syndrome. A long-term follow-up study Diabetes Care.

[55] Adami GA, Camerini G, Papadia MF, Catalano MF, Carlini F, Cordera R, Scopinaro N (2019). Type 2 diabetes remission and control in overweight and in mildly obese diabetic subjects at long-term follow-up after biliopancreatic diversion. Obes Surg.

