# Supplementary material for: Biliopancreatic Diversion (BPD), Long Common Limb Revisional Biliopancreatic Diversion (BPD + LCL–R), Roux-en-Y Gastric Bypass [RYGB] and Sleeve Gastrectomy (SG) mediate differential quantitative changes in body weight and qualitative modifications in body composition: a 5-year study
Source: Acta Diabetol. 2021 Aug 28;59(1):39–48. doi: 10.1007/s00592-021-01777-9 (PMC8758656; doi:10.1007/s00592-021-01777-9)
Supplement: Supplementary file 1 — Supplementary file1 (DOCX 1133 KB) [file 592_2021_1777_MOESM1_ESM.docx]

Supplemental Appendix

Biliopancreatic Diversion [BPD], Long Common Limb Revisional Biliopancreatic Diversion [LCL-R BPD], Roux-en-Y Gastric Bypass [RYGB], and Sleeve Gastrectomy [SG] mediate differential quantitative changes in body weight and qualitative modifications in body compositions.

Evolution of BMI, FM, FFM, TBW in the whole cohort of subjects undergoing bariatric surgery. BPD = biliopancreatic diversion; RYGB = gastric bypass; SG = sleeve gastrectomy; BPD+LCL-R = biliopancreatic diversion followed by revision (long common limb)


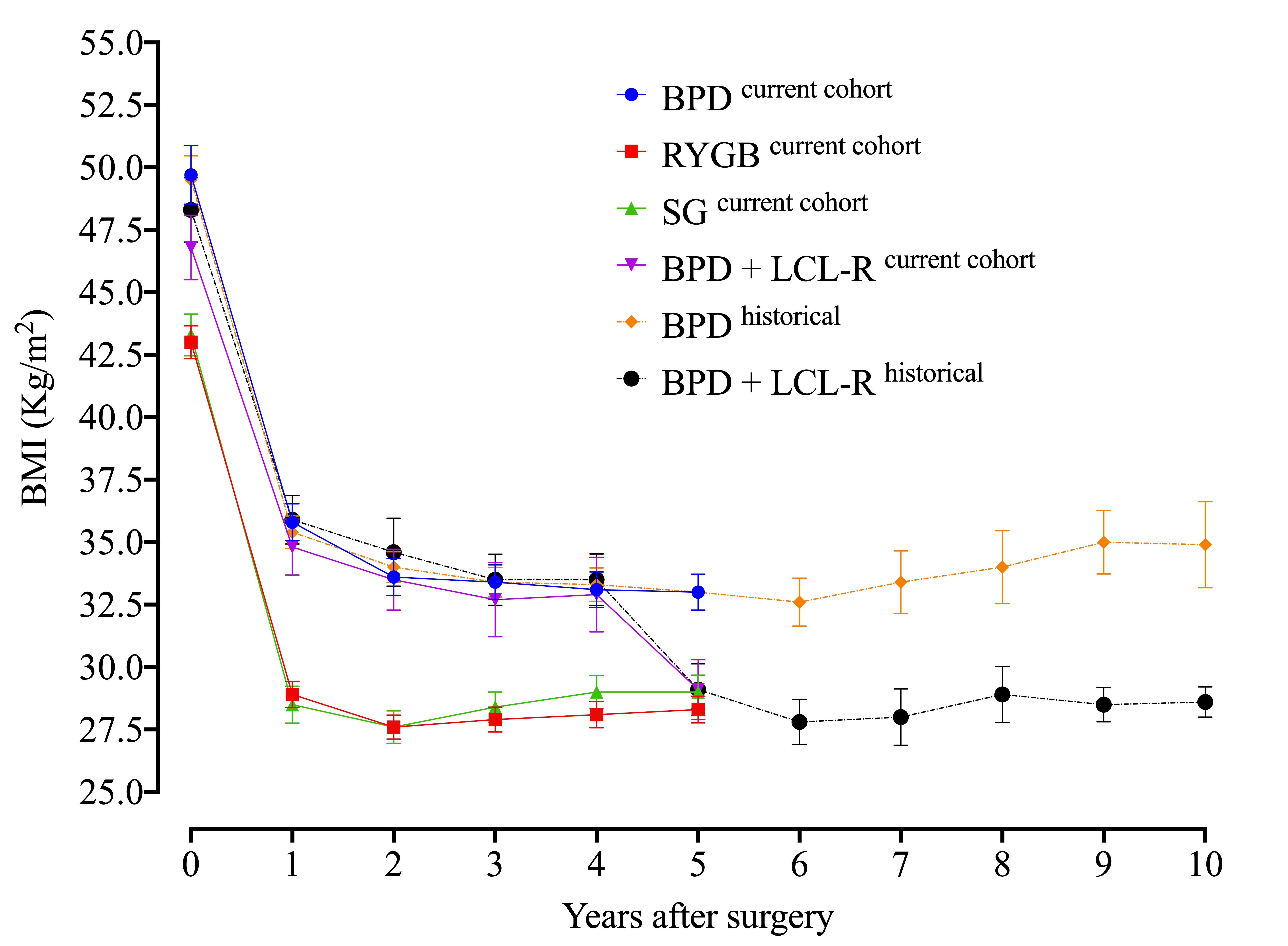


Fig. 1. BMI at various time intervals in the current cohort of patients compared with historical data (Ceriani V. et al, Obes Surg. 2017;27:1493-1500)

Table 1. Details of patients undergoing bariatric surgery at baseline. Absolute numbers and Means ± SD.

| Surgery | BPD | RYGB | SG | BPD+ LCLR | Significance (p) |
| --- | --- | --- | --- | --- | --- |
| Number (M/W) | 130 (29/101) | 135 (21/114) | 270 (48/222) | 30 (6/24) | NS |
| Age (years) | 43.5±10.85 | 43.1±9.29 | 44.6±10.41 | 42.7±10.87 | NS |
| BMI (kg/m^2^) | 49.3±8.22 | 44.9±10.37 * | 42.8±5.49 *§ | 47.5±6.81 | 0.001 |
| Weight (kg) | 129.9±24.35 | 118.4±19.46 * | 112.8±18.01 *§ | 126.3±23.36 | 0.001 |
| Excess weight (kg) | 63.9±21.86 | 52.5±15.34 * | 46.7±14.79 *§ | 59.9±18.86 | 0.001 |
| FM (kg) | 62.3±14.07 | 55.9±11.69 * | 52.6±10.49 *§ | 62.0±9.94 | 0.001 |
| FFM (kg) | 67.5±14.48 | 62.4±12.13 * | 60.2±11.96 * | 61.5±8.32 | 0.001 |
| FM (%) | 47.9±5.59 | 47.1±5.34 | 46.6±5.39 | 50.1±4.26 | NS |
| FFM (%) | 52.0±5.62 | 52.8±5.39 | 53.4±5.52 | 49.9±4.26 | NS |
| TBW (%) | 49.5±10.68 | 45.7±8.86 * | 44.1±8.62 * | 45.0±6.10 | 0.001 |

BPD= biliopancreatic diversion; RYGB = gastric bypass; SG = sleeve gastrectomy; BPD + LCL = biliopancreatic diversion followed by elongation of the common limb and restriction of the gastric pouch (median interval 4 years) FM = fat mass; FFM = fat-free mass; TBW = total body water

* vs BPD and BPD + LCLR; § vs RYGB

Table 2. Body mass index (BMI, kg/m^2^) in the whole population.

Means ± SD, number of subjects

surgery | BMI 0 BMI 1y BMI 2y BMI 3y BMI 4y BMI 5y

---------+------------------------------------------------------------

BPD | 49.4 36.0 33.9 33.4 33.2 32.9

| 8.23 5.46 5.25 5.04 5.39 5.37

| 130 130 130 84 57 56

---------+------------------------------------------------------------

RYGB | 44.9* 29.6*§ 28.3*§ 28.3*§ 28.1*§ 28.3*

| 5.37 4.58 4.48 4.17 3.68 3.71

| 135 135 135 91 48 48

---------+------------------------------------------------------------

SG | 42.8*§ 28.5*§ 28.1*§ 28.5*§ 29.0*§ 29.0*

| 5.49 4.42 4.32 4.50 4.55 4.59

| 270 270 270 115 46 46

---------+-----------------------------------------------------------

BPD+LCLR| 46.8 34.8 33.5 32.7 32.7 29.1#

| 7.07 6.15 6.66 8.18 7.24 6.58

| 30 30 30 30 30 30

* BPD vs RYGB and vs SG

§ BPD+LCL-R vs RYGB and vs SG

# BPD vs BPD+LCL-R

Table 3. Fat mass (FM, kg) in the whole cohort of subjects.

Means ± SD, number of subjects

surgery | FM 0 FM 1y FM 2y FM 3y FM 4y FM 5y

---------+------------------------------------------------------------

BPD | 62.2 34.9 31.3 29.5 29.2 28.9

| 13.98 10.19 10.26 9.53 9.05 9.55

| 130 130 130 84 57 56

---------+------------------------------------------------------------

RYGB | 55.9* 24.4*§ 22.3*§ 24.3* 22.5* 22.3*

| 11.69 9.40 9.14 15.64 8.15 7.24

| 135 135 135 91 48 48

---------+------------------------------------------------------------

SG | 52.6*§ 23.8*§ 23.5*§ 24.5*§ 26.0 26.0

| 10.49 9.14 8.75 9.11 10.29 10.17

| 270 270 270 115 46 46

---------+------------------------------------------------------------

BPD+LCLR| 62.0 33.4 31.0 30.4 30.2 22.9#

| 9.94 12.29 13.66 15.36 10.83 8.99

| 30 30 30 30 30 30

* BPD vs RYGB and vs SG

§ BPD+LCL-R vs RYGB and vs SG

# BPD vs BPD+LCL-R

Table 4. Fat-free mass (FFM, kg) in the whole cohort of subjects.

Means ± SD, number of subjects

surgery | FFM 0 FFM 1y FFM 2y FFM 3y FFM 4y FFM 5y

---------+------------------------------------------------------------

BPD | 67.6 59.7 58.1 57.6 57.3 56.5

| 14.53 11.04 10.92 11.23 10.92 10.06

| 130 130 130 84 57 56

---------+------------------------------------------------------------

RYGB | 62.4* 53.7*§ 52.2*§ 51.9* 50.8* 51.2

| 12.13 10.17 10.23 9.30 8.77 8.25

| 135 135 135 91 48 48

---------+------------------------------------------------------------

SG | 60.2* 51.5*§ 50.8*§ 49.8*§ 50.4* 50.4*

| 11.96 9.51 9.45 8.67 9.52 9.47

| 270 270 270 115 46 46

---------+------------------------------------------------------------

BPD+LCLR| 61.5 58.9 57.2 54.8 54.4 52.5

| 8.32 11.18 11.25 8.90 9.45 8.89

| 30 30 30 30 30 30

* BPD vs RYGB and vs SG

§ BPD+LCL-R vs RYGB and vs SG

Table 5. Total body water (TBW) in the whole cohort of subjects.

Means ± SD, number of subjects

surgery | TBW 0 TBW y1 TBW 2y TBW 3y TBW 4y TBW 5y

---------+------------------------------------------------------------

BPD | 49.5 43.6 42.5 42.2 41.9 41.4

| 10.72 8.12 8.01 8.23 8.03 7.40

| 130 130 130 84 57 56

---------+------------------------------------------------------------

RYGB | 45.7* 39.3*§ 38.5*§ 38.0* 37.2* 37.5*

| 8.86 7.44 7.36 6.81 6.42 6.03

| 135 135 135 91 48 48

---------+------------------------------------------------------------

SG | 44.1* 37.8*§ 37.4*§ 36.5*§ 36.9* 36.9*

| 8.62 6.95 7.05 6.36 6.99 6.95

| 270 270 270 115 46 46

---------+------------------------------------------------------------

BPD+LCLR| 45.0 42.9 41.9 40.1 40.1 38.5

| 6.10 8.40 8.20 6.45 6.92 6.51

| 30 30 30 30 30 30

* BPD vs RYGB and vs SG

§ BPD+LCL-R vs RYGB and vs SG

Table 6. Percent weight loss (EBWL%) in the whole cohort of subjects.

Means ± SD, number of subjects

surgery | ewl1 ewl2 ewl3 ewl4 ewl5

---------+--------------------------------------------------

BPD | 56.1 64.0 66.5 67.7 68.1

| 13.69 17.02 17.33 17.78 18.51

| 130 130 84 57 56

---------+--------------------------------------------------

RYGB | 78.8*§ 84.9*§ 83.9*§ 82.5* 81.7*

| 20.45 21.58 21.15 20.73 20.94

| 135 135 91 48 48

---------+--------------------------------------------------

SG | 83.3*§ 84.7*§ 82.2*§ 82.3* 78.6*

| 20.84 20.82 21.39 24.31 24.63

| 270 270 115 46 46

---------+--------------------------------------------------

BPD+LCLR| 54.9 60.4 64.9 79.0 81.2#

| 19.12 20.26 28.51 24.31 23.20

| 30 30 30 30 30

* BPD vs RYGB and vs SG

§ BPD+LCL-R vs RYGB and vs SG

# BPD vs BPD+LCL-R

**Differences between women and men at baseline and at various time intervals**

**Whole population**

Table 7. Body mass index (BMI, kg/m^2^) in the whole cohort of subjects.

Means ± SD, number of subjects

sex | bmi bmi1 bmi2 bmi3 bmi4 bmi5

---------+------------------------------------------------------------

women | 44.8362 30.77375 29.65054 30.02672 30.29662 30.22095

| 6.712974 5.814059 5.505111 5.569289 5.680728 5.53034

| 461 461 461 262 148 148

---------+------------------------------------------------------------

men | 45.78365 31.12885 30.50577 30.52414 30.03333 29.38125

| 7.144556 5.409658 4.661843 5.116067 4.895066 4.519737

| 104 104 104 58 33 32

Table 8. Fat mass (FM, kg) in the whole cohort of subjects.

Means ± SD, number of subjects

sex | fm fm1 fm2 fm3 fm4 fm5

---------+------------------------------------------------------------

women | 55.95495 27.78065 25.99196 27.14903 26.74041 26.52466

| 11.86753 10.49368 10.16337 12.44227 9.509673 9.1815

| 444 460 460 259 148 148

---------+------------------------------------------------------------

men | 55.47475 23.59712* 22.61553* 21.91091* 21.76667* 20.45313*

| 14.2732 11.15499 9.762009 9.831613 9.891526 8.902518

| 99 104 103 55 33 32

* significant vs women

Tab 9. Change of fat mass (Δ FM) in the whole cohort of subjects.

Means ± SD, number of subjects

sex | Δfm1 Δfm2 Δfm3 Δfm4 Δfm5

---------+---------------------------------------------------

women | 28.4 30.15811 28.89303 29.59231 29.65385

| 8.868057 10.72573 12.90575 11.2454 11.45596

---------+---------------------------------------------------

men | 32.09495* 32.86122* 33.66226* 32.43214 31.84444

| 11.53643 12.83878 15.1803 14.05089 14.18844

* significant vs women

Table 10. Fat free mass (FFM, kg) in the whole cohort of subjects.

Means ± SD, number of subjects

sex | ffm ffm1 ffm2 ffm3 ffm4 ffm5

---------+------------------------------------------------------------

women | 57.95586 50.48978 49.34652 49.52857 49.63904 49.60616

| 7.717822 6.334926 6.14446 6.231406 6.347765 6.093719

| 444 460 460 259 148 148

---------+------------------------------------------------------------

men | 83.09798* 71.24712* 70.06019* 68.70909* 68.8* 67.79688*

| 11.61663 9.338291 9.203903 9.693684 9.128321 8.049343

| 99 104 103 55 33 32

* significant vs women

Tab 11. Change of fat free mass (Δ FFM) in the whole cohort of subjects.

Means ± SD, number of subjects

sex | Δffm1 Δffm2 Δffm3 Δffm4 Δffm5

---------+---------------------------------------------------

women | 7.64009 8.770946 9.10041 9.9 9.770769

| 4.240482 4.683595 5.125716 6.460446 6.583376

---------+---------------------------------------------------

men | 12.17677* 13.37449* 14.83208* 12.81429* 13.14074*

| 5.637101 6.718728 9.425615 5.526014 5.565917

* significant vs women

Table 12. Total body water (TBW) in the whole cohort of subjects.

Means ± SD, number of subjects

sex | tbw tbw1 tbw2 tbw3 tbw4 tbw5

---------+------------------------------------------------------------

women | 42.48468 36.98283 36.26674 36.26834 36.34384 36.32603

| 5.539347 4.625602 4.478178 4.566622 4.665973 4.482196

| 444 460 460 259 148 148

---------+------------------------------------------------------------

men | 60.92323* 52.07404* 51.58738* 50.29273* 50.38182* 49.64688*

| 8.481069 7.015143 6.474076 7.089503 6.677876 5.884478

| 99 104 103 55 33 32

* significant vs women

**Differences between women and men at baseline and at various time intervals**

**Patients followed yearly for 5 years**

Table 13. Body mass index (BMI, kg/m^2^) in 180 subjects with yearly examinations.

Means ± SD, number of subjects

sex | bmi bmi1 bmi2 bmi3 bmi4 bmi5

---------+------------------------------------------------------------

women | 45.84047 32.01284 30.38176 30.50541 30.29662 30.22095

| 7.397547 6.199641 5.918931 5.835299 5.680728 5.53034

| 148 148 148 148 148 148

---------+------------------------------------------------------------

men | 45.58438 31.7375 30.74687 30.675 29.66563 29.38125

| 7.147935 5.64828 5.166329 5.407462 4.486511 4.519737

| 32 32 32 32 32 32

Table 14. Fat mass (FM, kg) in 180 subjects with yearly examinations.

Means ± SD, number of subjects

sex | fm fm1 fm2 fm3 fm4 fm5

---------+------------------------------------------------------------

women | 56.62214 29.19864 26.34966 27.11034 26.74041 26.52466

| 12.125 10.731 10.38283 10.43118 9.509673 9.1815

| 148 148 148 148 148 148

---------+------------------------------------------------------------

men | 53.3037 23.96562* 21.75484* 21.4069* 20.99063* 20.45313*

| 14.76351 12.59538 11.21169 9.458893 8.971257 8.902518

| 32 32 32 32 32 32

* significant vs women

Tab 15. Change of fat mass (Δ FM) in 180 subjects with yearly examinations.

Means ± SD, number of subjects

sex | deltafm1 deltafm2 deltafm3 deltafm4 deltafm5

---------+---------------------------------------------------

women | 28.01527 30.89008 29.86231 29.59231 29.65385

| 8.794994 10.6118 11.10076 11.2454 11.45596

---------+---------------------------------------------------

men | 30.06667 31.63846 31.35185 31.95926 31.84444

| 12.71701 12.94431 13.89639 14.08967 14.18844

Table 16. Fat free mass (FFM, kg) in 180 subjects with yearly examinations.

Means ± SD, number of subjects

sex | ffm ffm1 ffm2 ffm3 ffm4 ffm5

---------+------------------------------------------------------------

women | 59.34809 51.63946 50.39252 50.15862 49.63904 49.60616

| 8.950731 6.314178 6.131963 6.399225 6.347765 6.093719

| 148 148 148 148 148 148

---------+------------------------------------------------------------

men | 81.47037* 71.5625* 70.48387* 69.27586* 68.22500* 67.79688*

| 8.095336 8.647123 8.780664 8.235362 8.645902 8.049343

| 32 32 32 32 32 32

* significant vs women

Tab 17. Change of fat free mass (Δ FFM) in 180 subjects with yearly examinations.

Means ± SD, number of subjects

sex | Δffm1 Δffm2 Δffm3 Δffm4 Δffm5

---------+---------------------------------------------------

women | 8.158015 9.375572 9.585385 9.9 9.770769

| 5.291749 5.747884 6.203014 6.460446 6.583376

---------+---------------------------------------------------

men | 11.04444* 12.11154* 12.55556* 12.6963* 13.14074+

| 5.005715 5.672976 5.481039 5.595223 5.565917

* significant vs women

Table 18. Total body water (TBW) in 180 subjects with yearly examinations.

Means ± SD, number of subjects

sex | tbw tbw1 tbw2 tbw3 tbw4 tbw5

---------+------------------------------------------------------------

women | 43.36336 37.77755 36.9034 36.73448 36.34384 36.32603

| 6.349975 4.636496 4.488698 4.687015 4.665973 4.482196

| 148 148 148 148 148 148

---------+------------------------------------------------------------

men | 59.83704* 52.4125* 51.62581* 50.72414* 49.95938* 49.64688*

| 6.113865 6.289046 6.373956 5.972296 6.320875 5.884478

| 32 32 32 32 32 32

* significant vs women
